# Response to Letter to the Editor on “The associations among platelet count, mean platelet volume, and erectile dysfunction: an observational and Mendelian randomization study”

**DOI:** 10.1093/sexmed/qfaf016

**Published:** 2025-04-06

**Authors:** Jingxuan Peng, Jinshun An, Yuxing Chen, Jun Zhou, Boyu Xiang

**Affiliations:** Department of Urology, Xiangya Hospital, Central South University, Changsha 410000, China; Department of Urology, First Affiliated Hospital of Jishou University, Jishou 416000, China; Department of Urology, Xiangya Hospital, Central South University, Changsha 410000, China; Department of Biology, High School Attached to Hunan Normal University, Changsha 410000, China; Department of Urology, First Hospital of Hunan University of Chinese Medicine, Changsha 410000, China; Department of Urology, Xiangya Hospital, Central South University, Changsha 410000, China

To the Editor,

We are pleased to see that our article has received recognition and has sparked significant discussions.^1^ We appreciate their interest in our work and their efforts to further strengthen the scientific discourse in this area. To further foster this meaningful exchange, we will respond thoughtfully to each of the points raised.

Firstly, regarding the consideration of potential confounders in our Mendelian randomization (MR) analysis, we acknowledge the importance of addressing lifestyle factors, comorbidities, and environmental influences. While our study focused on the primary associations between platelet parameters and erectile dysfunction (ED), we agree that these factors could play a role in the underlying mechanisms. However, it is important to note that MR is designed to minimize confounding by leveraging genetic variants as instrumental variables. Although the random allocation of genetic variants theoretically controls for confounding factors, in reality, some genetic variants may still be associated with confounders such as environmental factors and lifestyle. These associations may arise through complex gene–environment interactions, making it difficult to fully satisfy the no-confounding assumption. As mentioned, lifestyle factors (e.g., diet, physical activity), comorbidities, and environmental influences are all subject to complex gene–environment interactions, making it difficult to find strong instrumental variables to control these potential confounders.^2^ Gene–environment interactions remain an unresolved issue that requires further exploration in future research.^3^

Secondly, we appreciate the suggestion to explore multivariate or mediator MR analysis to better understand the interactive effects between platelet count (PC) and mean platelet volume (MPV). We actually recommend conducting mediator analysis on clinical data and GWAS data separately to identify more clinically meaningful mediators. Future research should consider incorporating such methods to elucidate the complex interplay between these platelet parameters and their potential synergistic effects on ED development, which would indeed enhance the robustness of the findings and provide valuable insights into the pathophysiology of ED.

Lastly, as we mention in our discussion section, “future research should prioritize examining the associations between varying degrees of ED severity and platelet parameters.” We agree that stratifying ED by severity could offer a more nuanced understanding of the relationship between platelet parameters and different stages of the condition. However, the existing GWAS data did not stratify ED by severity, making it technically impossible to conduct subsequent analysis. Future research should consider this stratification to identify potential therapeutic targets for mild versus severe cases. This approach could help in developing more personalized interventions for patients with varying degrees of ED.

In conclusion, we thank the authors for their constructive feedback. Since our study combines clinical data with Mendelian randomization analysis, due to the limitations of current data and technology, we do not believe above suggestions will affect our research results. We believe that their suggestions will contribute to the advancement of research in this field and help in refining future studies.

## References

[1] Peng J, An J, Chen Y, Zhou J, Xiang B. The associations among platelet count, mean platelet volume, and erectile dysfunction: an observational and Mendelian randomization study. Sex Med. 2024;12(6):qfae093. 10.1093/sexmed/qfae093 PMC11723799 39801931

[2] Emdin CA, Khera AV, Kathiresan S. Mendelian randomization. JAMA. 2017;318(19):1925-1926. 10.1001/jama.2017.17219 29164242

[3] Virolainen SJ, VonHandorf A, Viel KCMF, Weirauch MT, Kottyan LC. Gene-environment interactions and their impact on human health. Genes Immun. 2023;24(1):1-11. 10.1038/s41435-022-00192-6 36585519 PMC9801363

